# Supplementary material for: Neighborhood-level disparities and subway utilization during the COVID-19 pandemic in New York City
Source: Nat Commun. 2021 Jun 17;12:3692. doi: 10.1038/s41467-021-24088-7 (PMC8211826; doi:10.1038/s41467-021-24088-7)

Neighborhood-level disparities and subway utilization during the COVID-19 pandemic in New York City

Supplementary Information

Carrión et al.

Supplementary Figure 1: Correlation plot of socioeconomic variables. Numbers and shading represent Kendall's tau correlation.

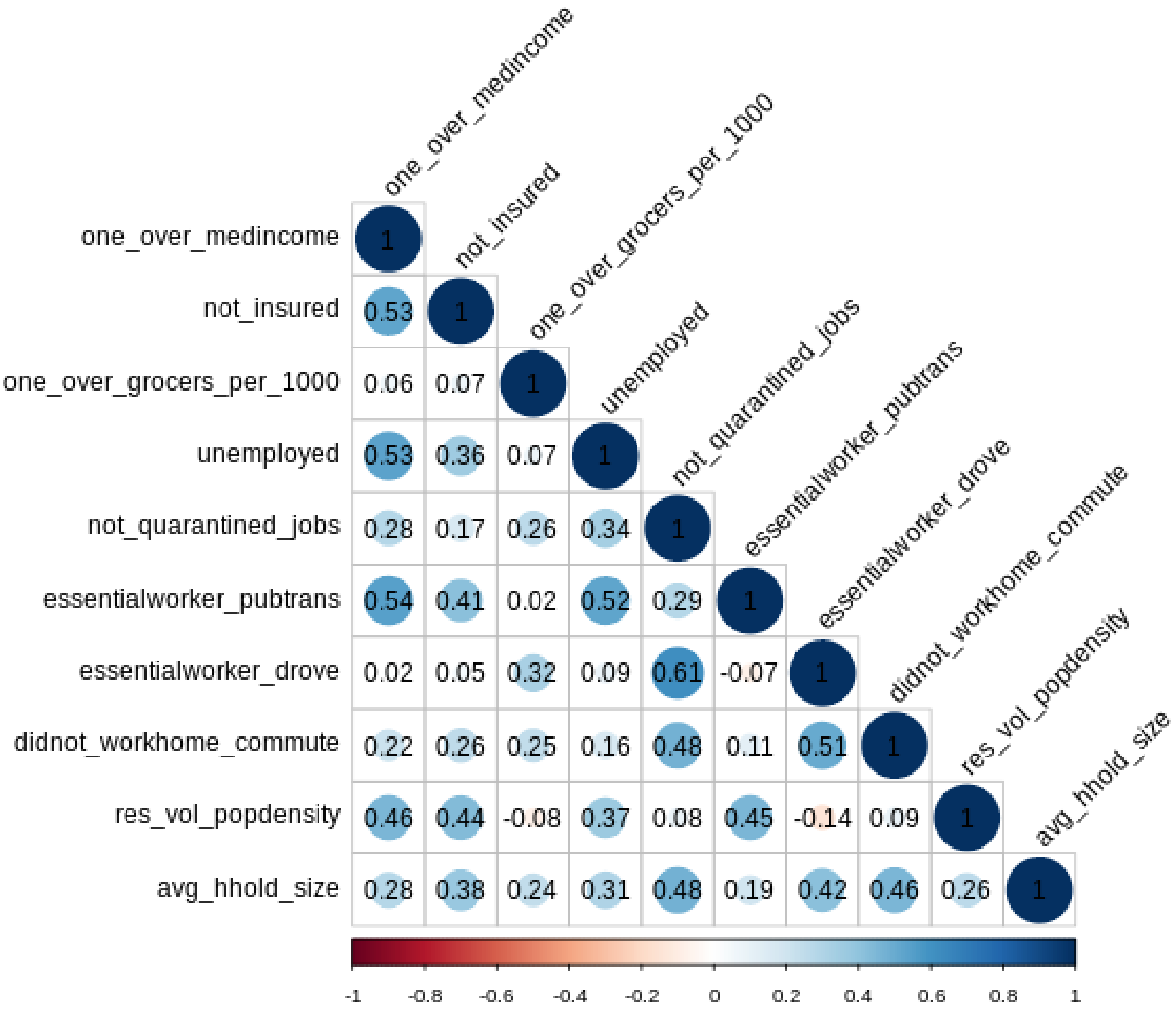

Supplementary Table 1: Kendall's tau correlations for social determinants with per capita cumulative SARS-CoV-2 infection incidence as of May 7, 2020 by NYC ZCTA.

Variable	Correlation	P value
1/median income	0.317	2.04×10^{-10}
Not insured (%)	0.231	3.01×10^{-6}
Unemployed (%)	0.296	1.25×10^{-7}
1/grocers per 1000 people	0.261	6.37×10^{-9}
Essential workers (%)	0.544	9.77×10^{-27}
Essential workers commuting via public transit (%)	0.174	5.56×10^{-4}
Essential workers commuting via car (%)	0.493	1.44×10^{-21}
People who do not work from home (%)	0.454	3.57×10^{-19}
Population density according to housing volume (people per cubic foot)	0.096	3.16×10^{-2}
Average household size (#)	0.467	7.70×10^{-20}

Supplementary Figure 2: Scatterplot of tests per capita and cumulative infection incidence. Unit of analysis is ZCTA (n=177). Line and grey ribbon are the natural cubic spline and 95% credible interval for the testing ratio (total tests divided by ZCTA population) from the BWQS negative binomial regression, holding the COVID-19 inequity index value constant at the median. Marginal histograms represent the distribution of the variable on each axis. Source data are provided as a Source Data file.

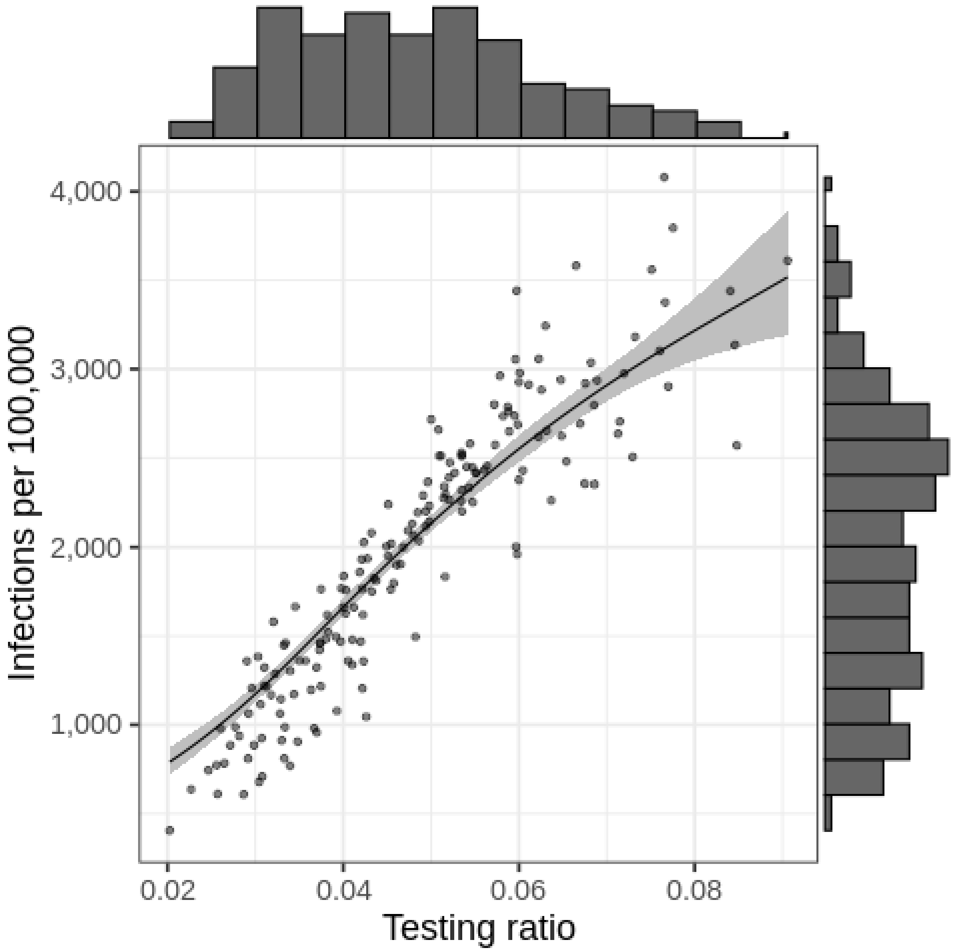

Supplementary Figure 3: Quantile-quantile plot of scaled residuals from the BWQS negative binomial regression model versus expected uniform distribution. The Kolmogorov-Smirnov test was two-sided. Source data are provided as a Source Data file.

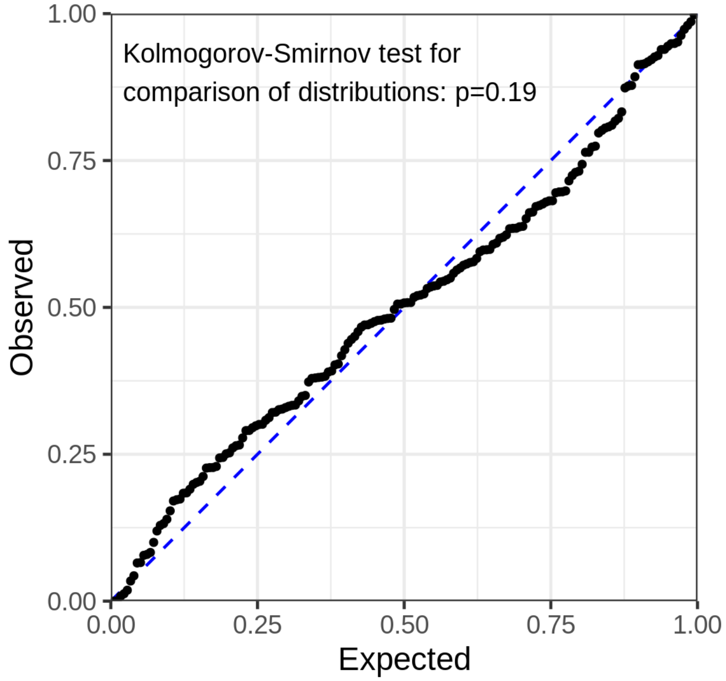

Supplementary Table 2: Parameter estimates, credible intervals, and diagnostics from main BWQS infections model

terms	mean	95% Credible Interval	median	Rhat	n_eff
Overdispersion	104.050	(81.448, 130.015)	103.552	0.999	1636
Intercept	6.261	(6.173, 6.352)	6.261	1.001	1643
COVID-19 inequity index	0.074	(0.063, 0.086)	0.074	1.000	1706
Testing ratio: spline term 1	0.984	(0.900, 1.072)	0.986	1.002	1905
Testing ratio: spline term 2	2.029	(1.809, 2.241)	2.029	1.001	1661
Testing ratio: spline term 3	1.123	(1.015, 1.233)	1.125	1.000	1792
1/ Median income	0.098	(0.009, 0.219)	0.093	0.999	1590
Uninsured	0.181	(0.067, 0.306)	0.179	0.999	1496
Unemployed	0.026	(0.001, 0.077)	0.021	1.000	1509
1/ Grocers per 1000	0.053	(0.002, 0.138)	0.046	1.000	1766
Essential Workers	0.062	(0.002, 0.181)	0.050	1.000	1767
Essential Worker: Public Transit	0.050	(0.002, 0.140)	0.043	0.999	1534
Essential Worker: Driving Commute	0.167	(0.033, 0.287)	0.171	0.999	1690
1/ Work from home	0.088	(0.010, 0.196)	0.083	1.002	1706
Population Density by Residential Volume	0.115	(0.018, 0.218)	0.113	1.000	1702
Average people per household	0.159	(0.038, 0.293)	0.159	1.000	1453

Supplementary Table 3: Ranks of social variable weights in posterior draws (17,500 with 10% thinning = 1,750).

Variable	Number of draws ranked highest	Percentage of draws ranked highest
1/median income	81	4.63%
Not insured (%)	686	39.20%
Unemployed (%)	0	0.00%
1/grocers per 1000 people	2	0.11%
Essential workers (%)	11	0.63%
Essential workers commuting via public transit (%)	0	0.00%
Essential workers commuting via car (%)	426	24.34%
People who do not work from home (%)	66	3.77%
Population density according to housing volume (people per cubic foot)	40	2.29%
Average household size (#)	438	25.03%

Supplementary Table 4: Comparison of model performance. All models are adjusted for the ZCTA-level testing ratio.

Regression type	**Root mean squared error** (infections per 100,000)	Kendall's τ
BWQS	187	0.872
Median income and proportion essential workers	291	0.825
Principal component	209	0.853

Supplementary Table 5: Comparison of BWQS models using ZCTA-level Census data versus tract-level using population-weighted median and 3rd quartiles.

Geographic unit and distribution measure	WAIC	Bayesian R^2	RMSE	Effect Estimate
ZCTA	2371	0.934	186	1.08
Tract, weighted median	2361	0.938	185	1.08
Tract, weighted 3rd quartile	2358	0.939	184	1.08

Supplementary Figure 4: Race/ethnic composition of ZCTAs that fall within various quantiles of the COVID-19 inequity index. Race/ethnic data from the 2018 ACS. NYC total demographic breakdown provided as reference. Source data are provided as a Source Data file.

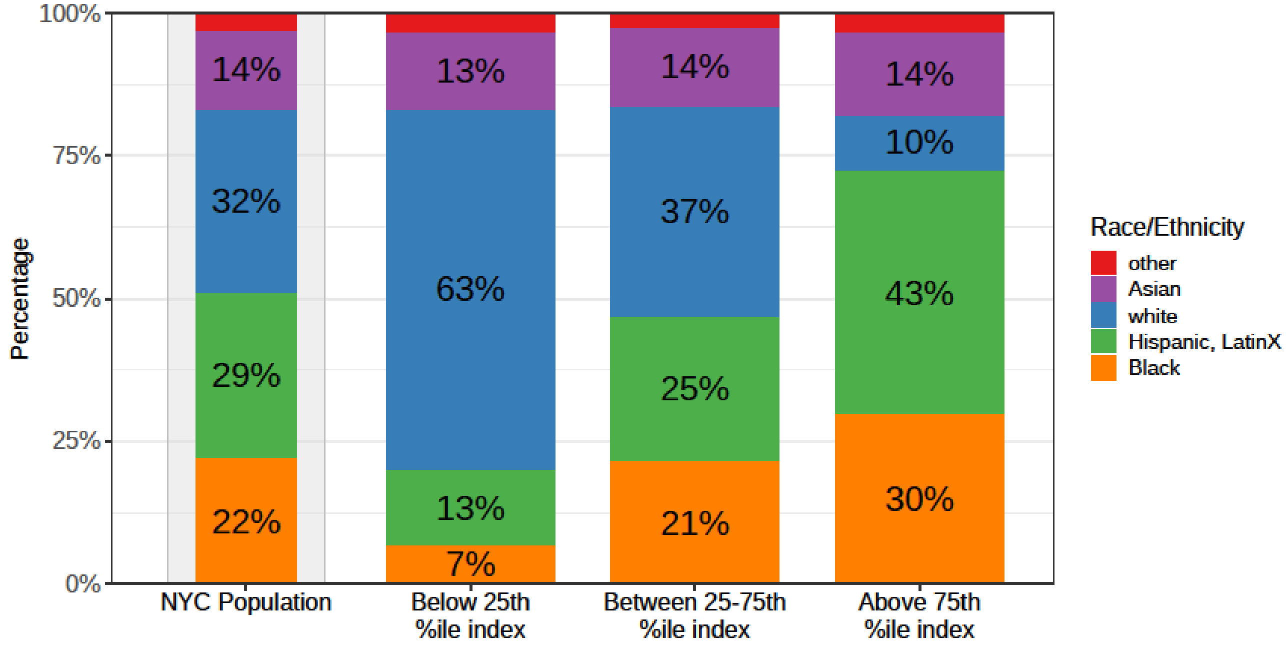

Supplementary Figure 5: United Hospital Fund (UHF) neighborhoods by population-weighted neighborhood COVID-19 inequity index. Six UHF neighborhoods excluded from the subway ridership analysis are indicated by asterisks. Dots represent subway stations with available data. UHF neighborhoods are colored as high (above median) or low (below median) COVID-19 inequity index. Light gray areas are non-residential locations. Base map and data from OpenStreetMap and OpenStreetMap Foundation. Source data are provided as a Source Data file.

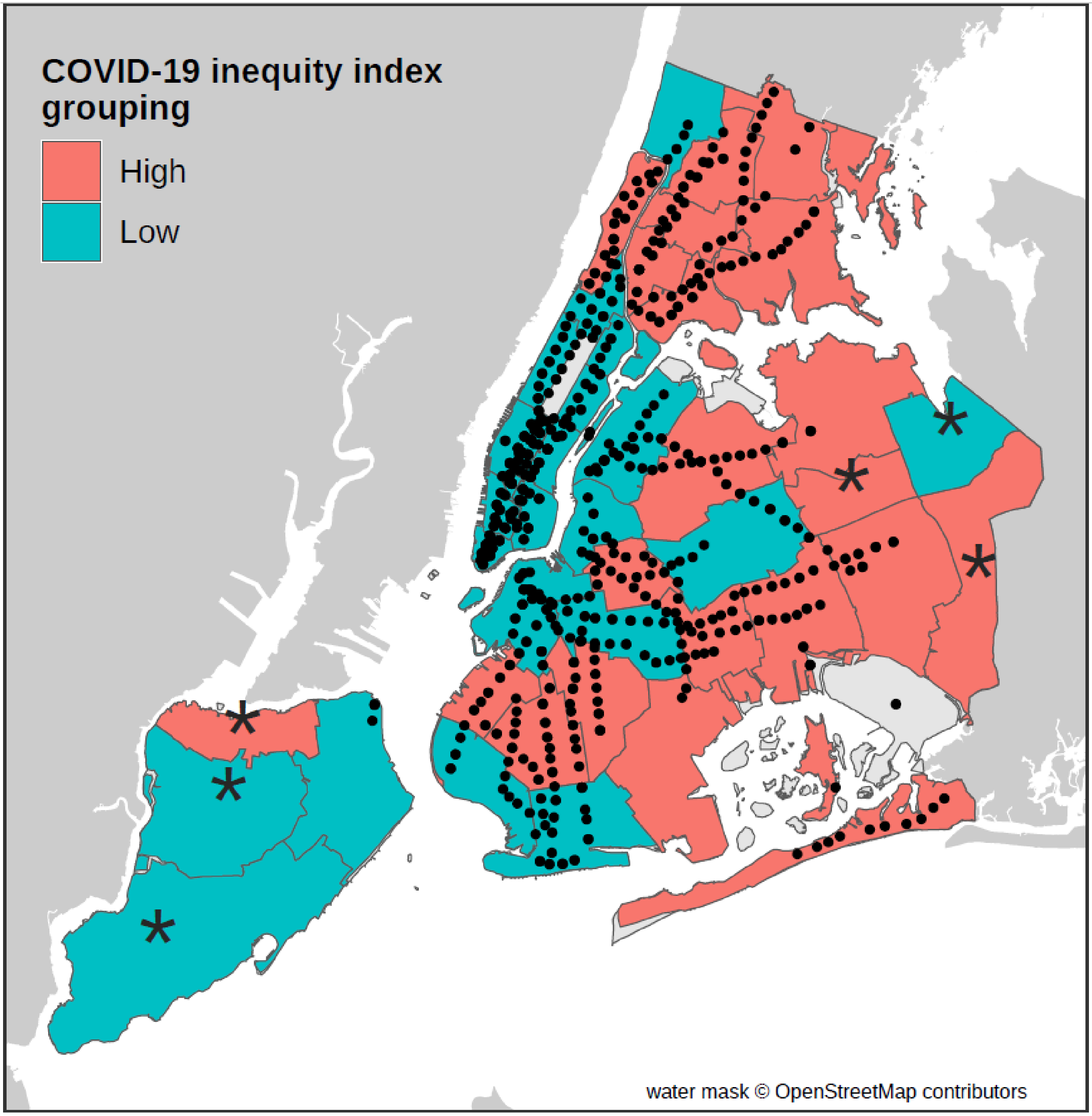

Supplementary Figure 6: Fit of the generalized Weibull equation curve on the citywide mean of United Hospital Fund-level ridership. Outlier on February 17th, a national holiday. Values above 1 indicate above-average ridership per our comparison with 2015-2019. 95% confidence intervals in gray bands. Source data are provided as a Source Data file.

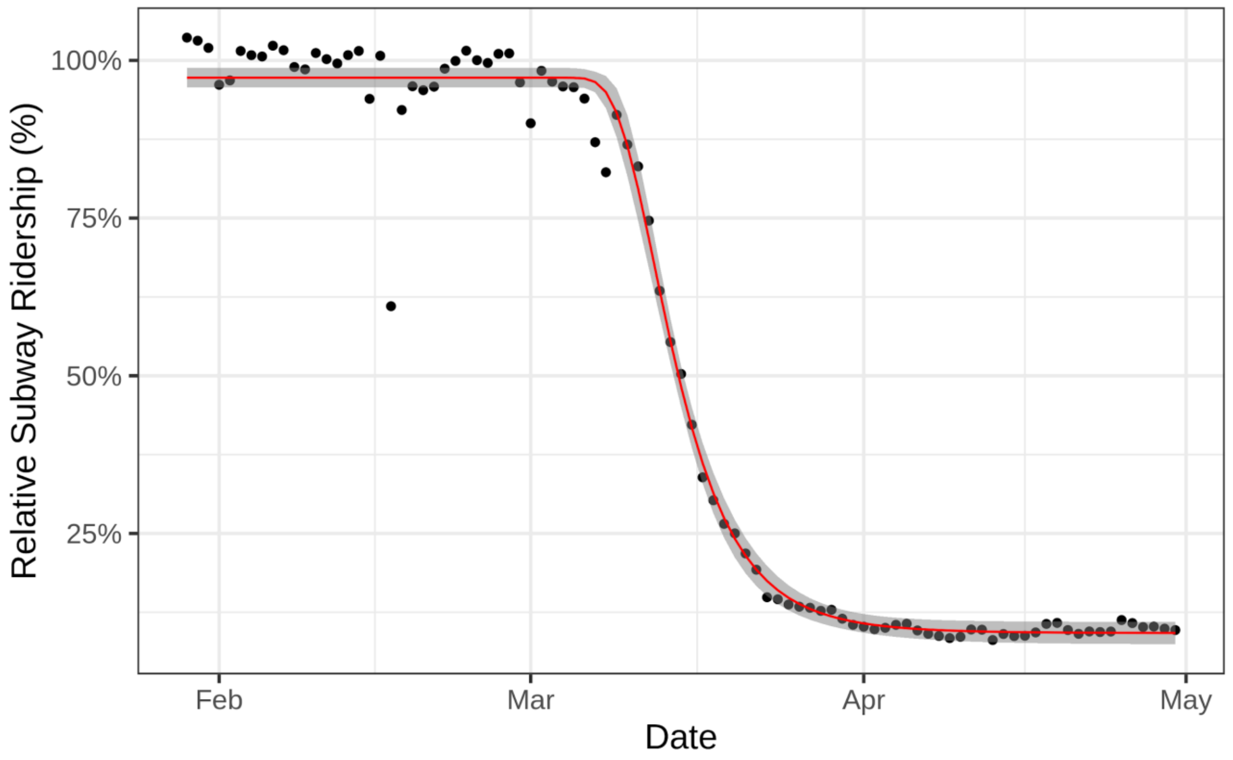

Supplementary Figure 7: Subway ridership trends by population-weighted COVID-19 inequity index at the United Hospital Fund neighborhood level. The nonlinear model was fitted using a generalized Weibull equation with three curves: high (>75%), mid (25%-75%) and low (<25%) COVID-19 inequity index at the UHF neighborhood level (n=36). Daily subway ridership is relative to 2015-2019. Dashed line represents the start of NYS on PAUSE social distancing policies. Ridership is shown between February 16, 2020 to April 30, 2020. Confidence intervals in gray bands. Source data are provided as a Source Data file.

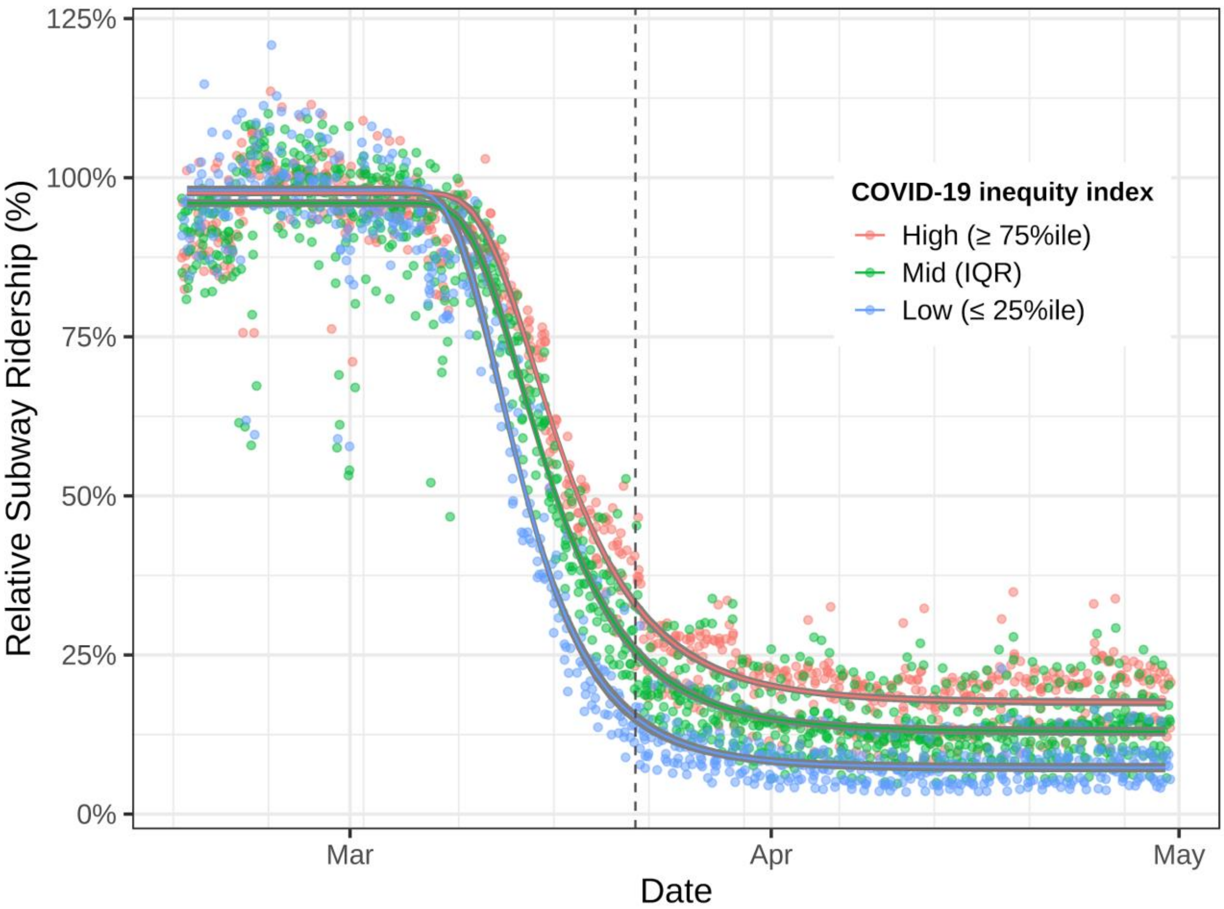

Supplementary Figure 8: Subway ridership trends by population-weighted COVID-19 inequity index at the zip code tabulation area level. The nonlinear model was fitted using a generalized Weibull equation with three curves: high (>75%), mid (25%-75%) and low (<25%) COVID-19 inequity index at the modified zip code tabulation area level (n=177). Daily subway ridership is relative to 2015-2019. Dashed line represents the start of NYS on PAUSE social distancing policies. Ridership is shown between February 16, 2020 to April 30, 2020. Confidence intervals in gray bands. Source data are provided as a Source Data file.

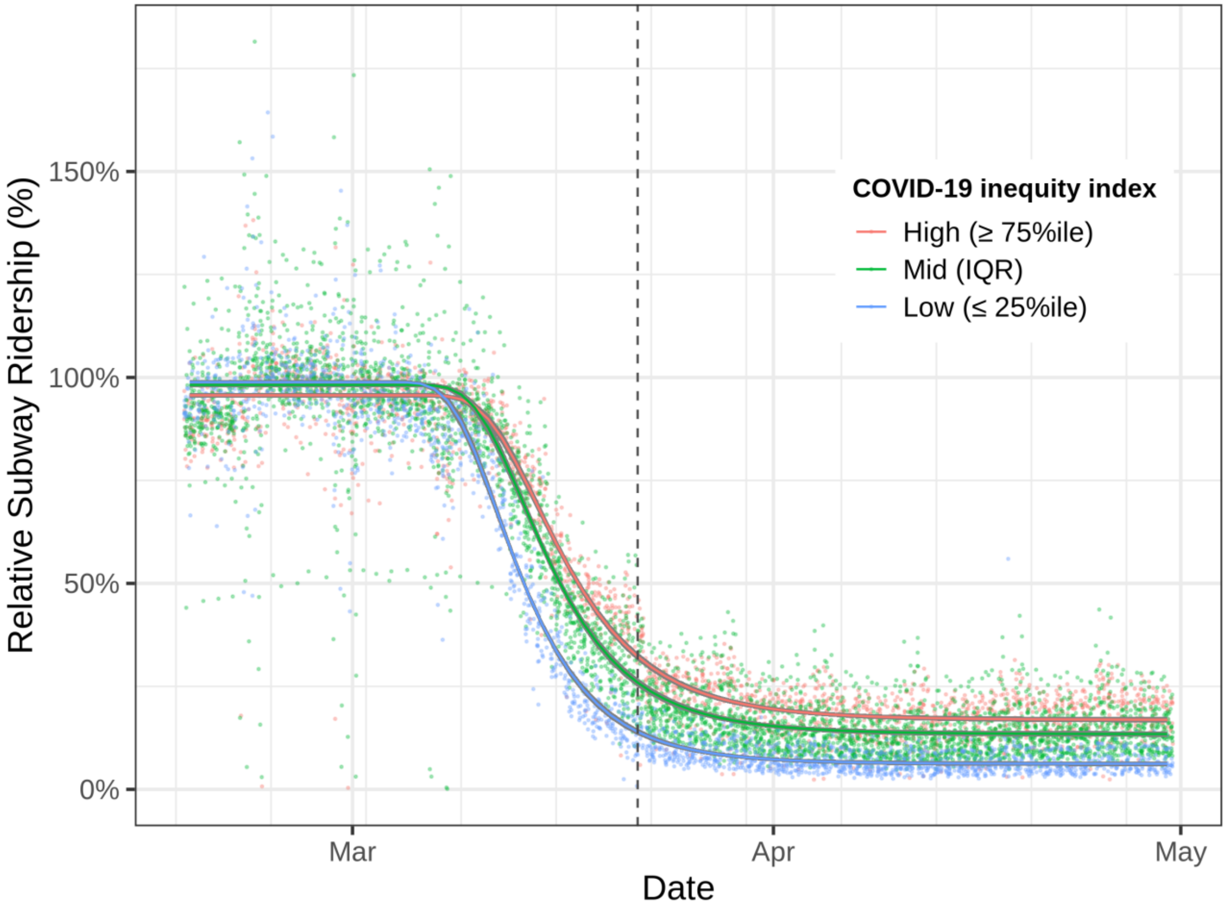

Supplementary Figure 9: Comparing MTA turnstile counts to Google mobility reports at the NYC borough level. MTA turnstile counts are available per subway station but only account for subway use. Google mobility reports are only available at the borough (county) level but capture all public transit use. Source data are provided as a Source Data file.

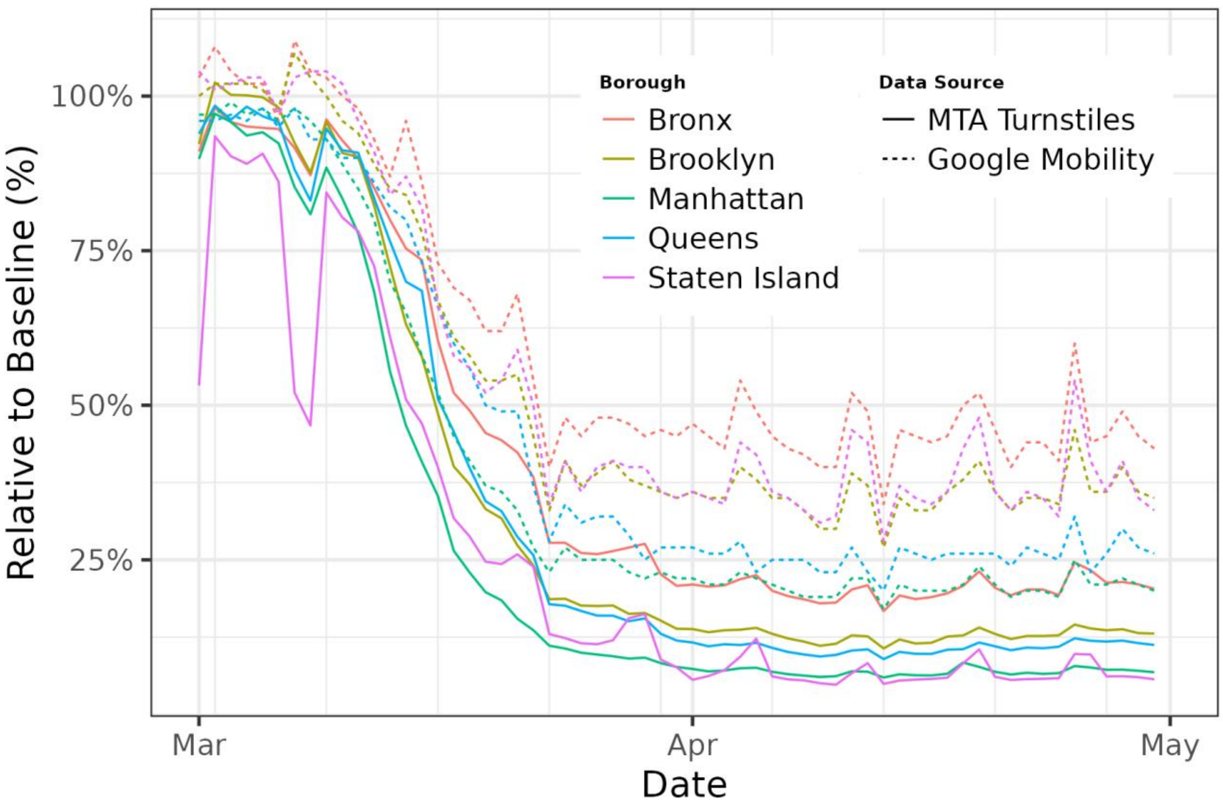

Supplementary Figure 10: Residuals from spatial filtered mortality analysis. Base map and data from OpenStreetMap and OpenStreetMap Foundation. Source data are provided as a Source Data file.

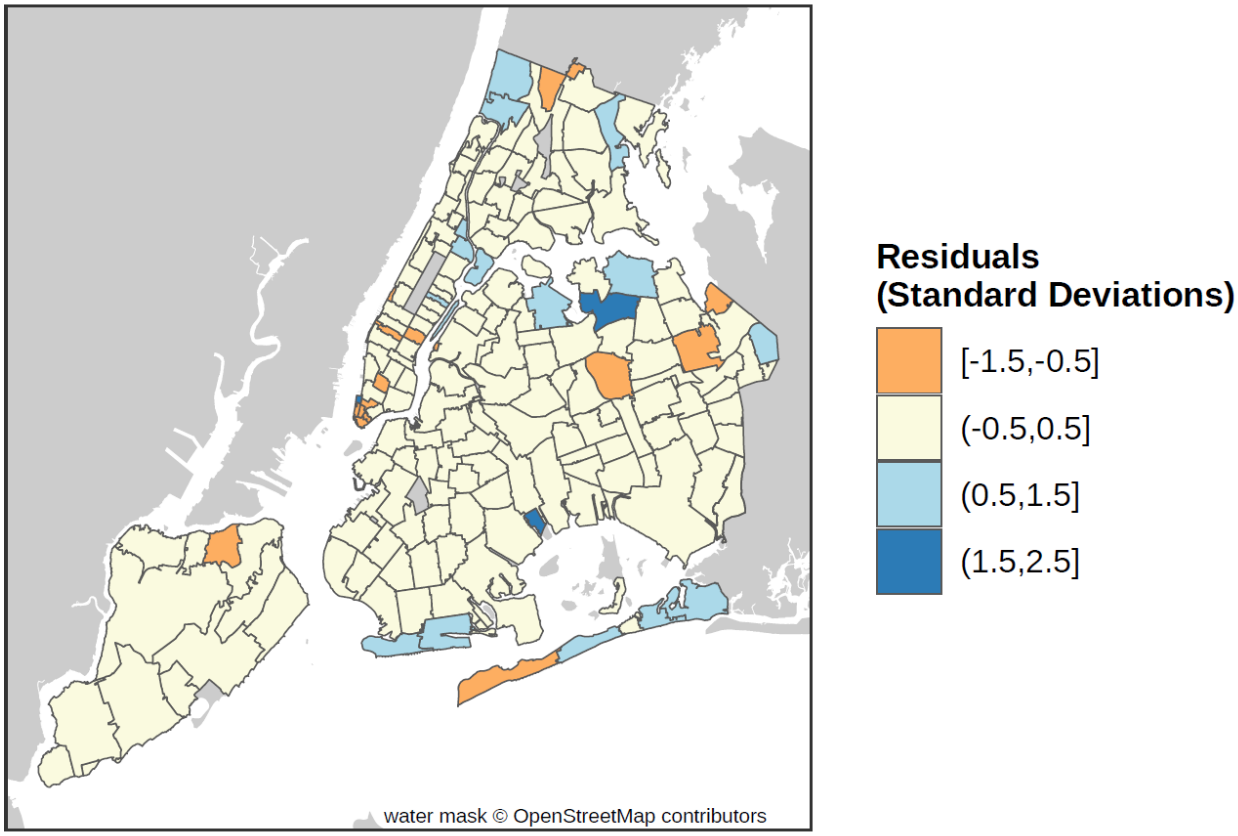

Supplementary Table 6: 2018 American Community Survey (ACS) 5-year estimate variables collected via *tidycensus* R package for ZIP code tabulation area (ZCTA) units.

ACS Variable Name	Short description	Explanation
B19013_001	Median income	Measure of neighborhood-level access to financial/economic resources
B06012_002	Federal Poverty Level (below 100%)	
B06012_003	Federal Poverty Level (between 100-150%)	
B23025_005	Unemployed	
B22003_002	Households with Supplementary nutrition assistance (SNAP)	Financial/economic resources and food access
B01003_001	Total population per ZCTA	For adjustment and crude population incidence calculations
B22003_001	Total households per ZCTA	To create household-based proportions
B27010_017	Uninsured (Under 19 years old)	Access to care and medical information/warnings
B27010_033	Uninsured (19-34 years old)	
B27010_050	Uninsured (35-64 years old)	
B27010_066	Uninsured (65+ years old)	
B08301_021	Work from home	Capacity to socially distance in early stages of pandemic
C24050_002	Employed by: Agricultural industry	Industries that are likely to employ essential workers
C24050_003	Employed by: Construction industry	
C24050_005	Employed by: Wholesale trade	
C24050_007	Employed by: Transportation industry	
C24050_011	Employed by: Education and Healthcare industry	
B08126_017	Employed by: Agriculture, Commute: personal car	Mode of transportation for industries employing essential workers
B08126_047	Employed by: Agriculture, Commute: Public Transit	
B08126_018	Employed by: Construction, Commute: personal car	
B08126_048	Employed by: Construction, Commute: Public Transit	
B08126_020	Employed by: Wholesale trade, Commute: personal car	
B08126_050	Employed by: Wholesale trade, Commute: Public Transit	
B08126_022	Employed by: Transportation, Commute: personal car	
B08126_052	Employed by: Transportation, Commute: Public Transit	
B08126_026	Employed by: Education/Healthcare, Commute: personal car	
B08126_056	Employed by: Education/Healthcare, Commute: Public Transit	

Supplementary Figure 11: Directed acyclic graph of proposed relationship between variables where x_1 through x_n are the social variables indicative of neighborhood disadvantage included in the COVID-19 inequity index. Light grey nodes are part of the theoretical pathway and relationship, but not adjusted for or explicitly included in the analyses.

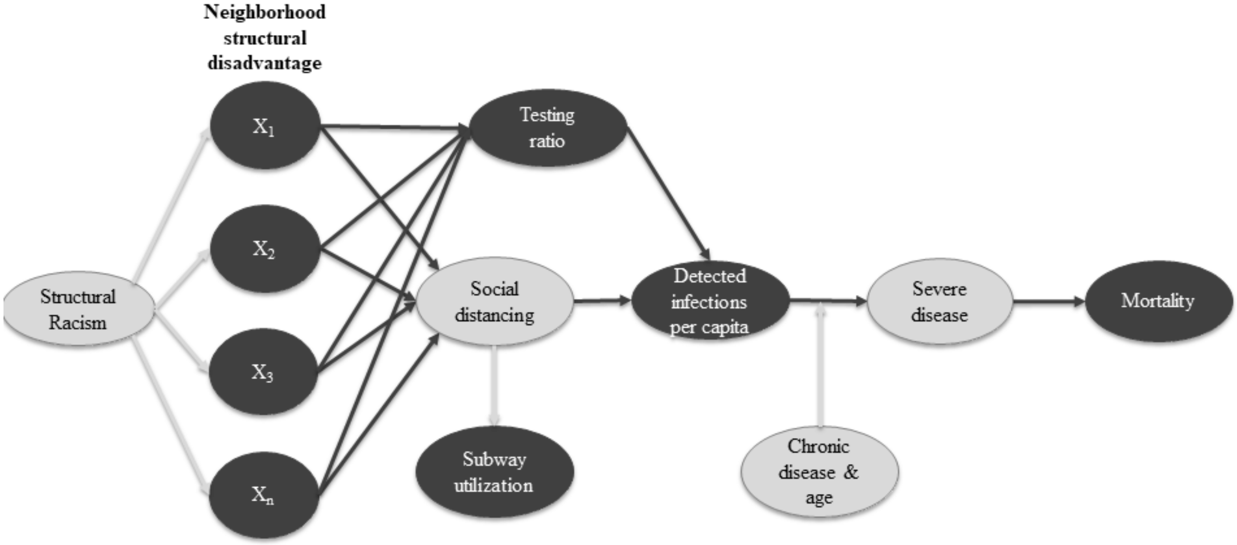

Supplement: Supplementary file 1 — Supplementary Information [file 41467_2021_24088_MOESM1_ESM.pdf]
