# Supplementary material for: Neighborhood-level disparities and subway utilization during the COVID-19 pandemic in New York City
Source: Nat Commun. 2021 Jun 17;12:3692. doi: 10.1038/s41467-021-24088-7 (PMC8211826; doi:10.1038/s41467-021-24088-7)
Supplement: Supplementary file 3 — Reporting Summary [file 41467_2021_24088_MOESM3_ESM.pdf]

nature research

Corresponding author(s): Daniel Carrión

Last updated by author(s): Mar 23, 2021

Reporting Summary

Nature Research wishes to improve the reproducibility of the work that we publish. This form provides structure for consistency and transparency in reporting. For further information on Nature Research policies, see our Editorial Policies and the Editorial Policy Checklist.

Statistics

For all statistical analyses, confirm that the following items are present in the figure legend, table legend, main text, or Methods section.

n/a	Confirmed	
☐	☒	The exact sample size (*n*) for each experimental group/condition, given as a discrete number and unit of measurement
☐	☒	A statement on whether measurements were taken from distinct samples or whether the same sample was measured repeatedly
☐	☒	The statistical test(s) used AND whether they are one- or two-sided<br>*Only common tests should be described solely by name; describe more complex techniques in the Methods section.*
☐	☒	A description of all covariates tested
☐	☒	A description of any assumptions or corrections, such as tests of normality and adjustment for multiple comparisons
☐	☒	A full description of the statistical parameters including central tendency (e.g. means) or other basic estimates (e.g. regression coefficient) AND variation (e.g. standard deviation) or associated estimates of uncertainty (e.g. confidence intervals)
☐	☒	For null hypothesis testing, the test statistic (e.g. *F*, *t*, *r*) with confidence intervals, effect sizes, degrees of freedom and *P* value noted<br>*Give P values as exact values whenever suitable.*
☐	☒	For Bayesian analysis, information on the choice of priors and Markov chain Monte Carlo settings
☒	☐	For hierarchical and complex designs, identification of the appropriate level for tests and full reporting of outcomes
☒	☐	Estimates of effect sizes (e.g. Cohen's *d*, Pearson's *r*), indicating how they were calculated

Our web collection on statistics for biologists contains articles on many of the points above.

Software and code

Policy information about availability of computer code

Data collection	All data was collected via government websites using the R statistical programming language
Data analysis	All data was analyzed using the R statistical programming language version 4.0.2, and the BWQS regression (including Hamilton-Monte Carlo algorithm) was implemented with rstan version 2.19.3.

For manuscripts utilizing custom algorithms or software that are central to the research but not yet described in published literature, software must be made available to editors and reviewers. We strongly encourage code deposition in a community repository (e.g. GitHub). See the Nature Research guidelines for submitting code & software for further information.

Data

Policy information about availability of data

All manuscripts must include a data availability statement. This statement should provide the following information, where applicable:

- Accession codes, unique identifiers, or web links for publicly available datasets
- A list of figures that have associated raw data
- A description of any restrictions on data availability

Census data were drawn from https://api.census.gov/data/ using the tidycensus package in R. NYC buildings data were drawn from https://www1.nyc.gov/assets/planning/download/zip/data-maps/open-data/nyc_pluto_20v3_csv.zip and https://data.cityofnewyork.us/api/geospatial/nqwf-w8eh?method=export&format=Shapefile. Zip code neighborhood definitions were accessed from https://www.health.ny.gov/statistics/cancer/registry/appendix/neighborhoods.htm. NYC COVID-19 testing and mortality data: https://raw.githubusercontent.com/nychealth/coronavirus-data/6d7c4a94d6472a9ffc061166d099a4e5d89cd3e3/tests-by-zcta.csv. United Hospital Fund shapefile: https://www1.nyc.gov/assets/doh/downloads/zip/uhf42_dohmh_2009.zip. NYC Boroughs shapefile: https://data.cityofnewyork.us/api/geospatial/tqmj-j8zm?method=export&format=Shapefile. Modified ZCTA shapefile: https://data.cityofnewyork.us/api/geospatial/pri4-ifjk?method=export&format=Shapefile. Food retailers in New York State: https://data.ny.gov/api/

views/9a8c-vfzj/rows.csv. Crosswalk table of ZCTAs to modified ZCTAs: https://raw.githubusercontent.com/nychealth/coronavirus-data/master/Geography-resources/ZCTA-to-MODZCTA.csv. Crosswalk table of ZCTA to Census Tracts: https://www.huduser.gov/portal/datasets/usps/ZIP_TRACT_062020.xlsx. Geocoding tool for New York State: https://gisservices.its.ny.gov/arcgis/rest/services/Locators/Street_and_Address_Composite/GeocodeServer/findAddressCandidates?f=json&maxLocations=1&SingleLine=.

Field-specific reporting

Please select the one below that is the best fit for your research. If you are not sure, read the appropriate sections before making your selection.

☒ Life sciences ☐ Behavioural & social sciences ☐ Ecological, evolutionary & environmental sciences

For a reference copy of the document with all sections, see nature.com/documents/nr-reporting-summary-flat.pdf

Life sciences study design

All studies must disclose on these points even when the disclosure is negative.

Sample size	Sample sizes were based on maximizing available data. Therefore, 174,614 unique positive COVID-19 cases across 177 modified zip-code tabulation areas (ZCTAs) were used for the development of the neighborhood infection risk score. 36 United Hospital Fund areas were used in our social distancing analysis. And 16,289 COVID-related deaths across 177 ZCTAs were used in our mortality analysis.
Data exclusions	We omitted outliers from our ridership data in the NYC Subway analysis depicted in Figure 5 and Table 1. This functionally only impacted one zip code, across 6 observations, which was a small fraction of the overall data. These outliers aligned with planned service changes on weekends in the Bronx, which means that service was either suspended or modified by the Metropolitan Transit Authority due to track or station work, and not differences in rider demand.
Replication	All analytical code is provided in our Zenodo repository.
Randomization	This was an retrospective epidemiological study, not an experimental study. Data were drawn from public datasets of COVID-19 cases and deaths, and we conducted associational analyses of neighborhood-level factors. These factors (such as housing density and food access) are not intervenable within the context of our study. For these reasons randomization was not feasible. Potential confounders were adjusted for, when relevant.
Blinding	This was an retrospective observational epidemiological study, not an experimental study. The study and analyses were entirely computational. Blinding in the context of a retrospective design with no assigned treatments was not feasible, because all of the events already occurred and the authors conducted all components of the research, including data analysis, interpretation of results, and manuscript preparation.

Reporting for specific materials, systems and methods

We require information from authors about some types of materials, experimental systems and methods used in many studies. Here, indicate whether each material, system or method listed is relevant to your study. If you are not sure if a list item applies to your research, read the appropriate section before selecting a response.

Materials & experimental systems

n/a	Involved in the study
☒	☐ Antibodies
☒	☐ Eukaryotic cell lines
☒	☐ Palaeontology and archaeology
☒	☐ Animals and other organisms
☒	☐ Human research participants
☒	☐ Clinical data
☒	☐ Dual use research of concern

Methods

n/a	Involved in the study
☒	☐ ChIP-seq
☒	☐ Flow cytometry
☒	☐ MRI-based neuroimaging
